# The Placenta—A New Source of Bile Acids during Healthy Pregnancy? First Results of a Gene Expression Study in Humans and Mice

**DOI:** 10.3390/ijms24119511

**Published:** 2023-05-30

**Authors:** Edgar Ontsouka, Mariana Schroeder, Linda Ok, Cathy Vaillancourt, Deborah Stroka, Christiane Albrecht

**Affiliations:** 1 Faculty of Medicine, Institute of Biochemistry and Molecular Medicine, University of Bern, Bühlstrasse 28, 3012 Bern, Switzerland; edgar.ontsouka@unibe.ch (E.O.); mariana.schroeder@unibe.ch (M.S.); 2 Institut National de la Recherche Scientifique (INRS), Centre Armand Frappier et Regroupement Intersectoriel de Recherche en Santé de l’Université du Quebec (RISUQ), 532 des Prairies, Laval, QC H7V1B7, Canada; linda.ok@inrs.ca (L.O.); cathy.vaillancourt@inrs.ca (C.V.); 3 Department of Visceral Surgery and Medicine, Inselspital, Bern University Hospital, University of Bern, Murtenstrasse 35, 3002 Bern, Switzerland; deborah.stroka@unibe.ch

**Keywords:** extrahepatic, bile acid synthesis, mice, human, pregnancy

## Abstract

Bile acids (BAs) are natural ligands for several receptors modulating cell activities. BAs are synthesized via the classic (neutral) and alternative (acidic) pathways. The classic pathway is initiated by CYP7A1/Cyp7a1, converting cholesterol to 7α-hydroxycholesterol, while the alternative pathway starts with hydroxylation of the cholesterol side chain, producing an oxysterol. In addition to originating from the liver, BAs are reported to be synthesized in the brain. We aimed at determining if the placenta potentially represents an extrahepatic source of BAs. Therefore, the mRNAs coding for selected enzymes involved in the hepatic BA synthesis machinery were screened in human term and CD1 mouse late gestation placentas from healthy pregnancies. Additionally, data from murine placenta and brain tissue were compared to determine whether the BA synthetic machinery is comparable in these organs. We found that *CYP7A1, CYP46A1,* and *BAAT* mRNAs are lacking in the human placenta, while corresponding homologs were detected in the murine placenta. Conversely, *Cyp8b1* and *Hsd17b1* mRNA*s* were undetected in the murine placenta, but these enzymes were found in the human placenta. *CYP39A1/Cyp39a1* and cholesterol 25-hydroxylase (*CH25H/Ch25h*) mRNA expression were detected in the placentas of both species. When comparing murine placentas and brains, *Cyp8b1* and *Hsd17b1* mRNAs were only detected in the brain. We conclude that BA synthesis-related genes are placentally expressed in a species-specific manner. The potential placentally synthesized BAs could serve as endocrine and autocrine stimuli, which may play a role in fetoplacental growth and adaptation.

## 1. Introduction

Bile acids (BAs) are synthesized from cholesterol mainly in hepatocytes through the classic (or neutral) and alternative (or acidic) pathways, which differ in their initial steps [1,2,3]. In short, the classic pathway is initiated by the microsomal enzyme cytochrome P450 (CYP) 7A1, which converts cholesterol to 7α-hydroxycholesterol. Depending on CYP8B1/Cyp8b1 activities, which induce the 12α-hydroxylation of the mentioned precursor, either chenodeoxycholic acid or cholic acid is produced [1]. On the other hand, the alternative pathway begins with hydroxylation of the side chain of cholesterol that produces an oxysterol. The latter is then hydroxylated at the 7-position by an oxysterol 7α-hydroxylase (CYP7B1/Cyp7b1 or CYP39A1/Cyp39a1). Depending on the organ, sterol 27-hydroxylase (CYP27A1/Cyp27a1, in the liver) and/or cholesterol 25-hydroxylase (CH25H/Ch25h, in the liver) and/or cholesterol 24-hydroxylase (CYP46A1/Cyp46a1; CH24H, in the liver and brain) initiate the alternative BA biogenesis. Synthetic steps downstream of the formation of 7α-hydroxylated intermediates are similar in the classic and alternative pathways [1,2,3]. 

Despite the original belief that solely the enterohepatic axis determines the serum BA levels [4], evidence suggests other potential sources (e.g., the brain [5,6]) that can influence the serum BA concentrations and provoke functional effects. In the context of pregnancy, previous studies have shown that serum BA levels are higher in pregnant compared to nonpregnant mice and women [7,8]. There seems to be no direct relationship between gestational age and BA concentrations [9,10]; however, in most of the reported studies, this aspect was not directly addressed. Interestingly, the BA gradient observed in healthy pregnancies [10,11,12] shows higher BA levels on the fetal than on the maternal side. This trend is reversed during intrahepatic cholestasis of the pregnancy (ICP) [13]. Hence, while the placenta seems to act protectively against maternal BAs in the pathologic condition of ICP, its role regarding BAs might be different during a healthy pregnancy. Although the placenta is an important steroidogenic organ capable of metabolizing large amounts of cholesterol and is also expressing BA transporters [14], the possibility that it may serve as an additional source of increased serum BA levels during pregnancy has never been investigated. Of note, the receptor-mediated activities of BAs modulate important cellular/tissue events, such as cell proliferation and apoptosis, which are all important for adequate placental and fetal development [15,16,17,18]. Interestingly, the presence of the BA metabotropic receptor TGR5 in the human placenta has been documented [19]. This also implies the possibility of autocrine/paracrine effects mediated by locally synthesized BA, which may induce the related signaling pathways in the placenta. 

We hypothesized that the placenta expresses the mRNA transcripts encoding the enzymes implicated in BA synthetic pathways and could therefore be an additional source of BAs. Hence, we analyzed the gene expression profiles of fifteen selected components of the classic and alternative BA synthetic pathways [1] in human term placentas and in murine placentas at gestation day 17.5. Of note, the mouse data reported in this study were generated from a deposited database and are not based on a new set of animals [20,21]. Our objectives were to determine: (i) the presence of mRNAs encoding enzymes of the classic and alternative BA synthetic pathways in human and murine placentas, (ii) the differences or similarities of this synthetic machinery in the placentas of both species, and (iii) if the BA synthetic pathway patterns in two extrahepatic organs (i.e., murine placenta and the hypothalamus) are comparable. 

## 2. Results and Discussion

**Figure 1:**
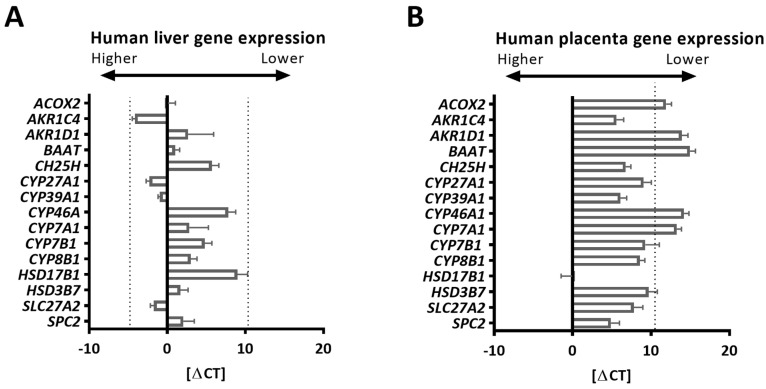
Expression of bile acid synthesis-related genes in the liver and placenta. (**A**,**B**) Using primers validated in the positive tissue (human liver, n = 3), the expression of selected bile acid synthesis-related genes in the human placenta was tested by real-time quantitative RT-PCR. The gene expression of bile acid-related genes in the liver was used as the positive control since the liver is a primary source of bile acids. Gene expression data are shown as delta Ct values (Ct of target gene—mean Ct of the reference genes). Given the mean Ct values of YWHAZ and ubiquitin (used as the reference genes) in the human placenta (24.9 ± 2.3) and liver (23.7 ± 0.7), the delta Ct value (0) equals the target Ct values of 24.9 (in the placenta) and 23.7 (in the liver), respectively. The expression of the determined genes ranged between the Ct values 19.2 (lowest Ct value) and 34.4 (highest Ct value) in the human liver. Thus, the latter value served for the evaluation of gene expression in the placenta and was defined as the cut-off value above which the gene expression (detected in at least 80% of the studied samples) was considered as lacking ((**B**), dashed line). A gender-balanced cohort of human placentas (n = 6 females, n = 6 males) was used.

All the tested human BA synthesis-related genes were detected in the human liver, with the highest Ct value of 34.4 (Table 1, Figure 1A). The Ct value represents the PCR amplification cycle number (“cycle threshold”) inversely proportional to the gene amount at which the detected fluorescent curve crosses the threshold in the linear part of the amplification curve. Therefore, to assess gene expression in the human placenta, this Ct value served as a cut-off value above which gene expression was considered as undetected (Figure 1B, dashed line). The demographic characteristics (including age and sex) of the liver donors are unknown. Therefore, it cannot be excluded that some of these parameters might have had an impact on the expression levels detected in liver tissue. Of note, the concentration of total BAs in gallbladder bile is reported to be significantly greater in women than in men [22]. Applying the criteria detailed in the Section 3.4., most BA synthesis-related genes were present in the human placenta (Figure 1B). 

**Figure 2:**
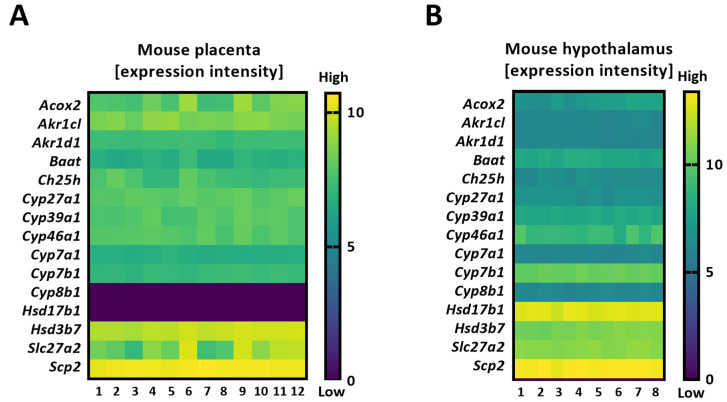
Heatmaps detailing the expression intensity of the genes involved in bile acid biosynthesis in the mouse placenta (based on RNA-seq) (**A**) and mouse hypothalamus (Agilent array) (**B**), as published elsewhere [20,21].

*CYP7A1* was undetected in human placentas, implying the absence of the classical pathway (Table 1 and Figure 1B). In contrast, its homolog was present in murine placentas (Figure 2A). Enzymes driving the initial steps of the alternative BA synthesis, *CYP27A1/Cyp27a1* and *CH25H*/*Ch25h* [1,2,3], were expressed in both species (Figure 1B and Figure 2A), suggesting these pathways are conserved in the placenta.

*CYP46A1,* another alternative BA synthesis pathway ([5]), was undetectable in the human placenta, while it was expressed in human livers (Figure 1A,B and Table 1). *Cyp46a1* was also found in extrahepatic tissues in mice (Figure 2A,B), suggesting a species- and tissue-specific importance of the CYP46A1/Cyp46a1-related pathway. *CYP39A1*/*Cyp39a1,* an oxysterol 7α-hydroxylase and critical enzyme required in the alternative pathway, was expressed in the placentas of both species (Figure 1B and Figure 2A), as well as in the murine hypothalamus (Figure 2B). The fact that *Cyp8b1 and Hsd17b1* were expressed in mouse hypothalamic tissue but not in the placenta (Figure 2A,B) proposes that the alternative BA biosynthesis pathway in extrahepatic tissues may not be analogous. 

Another interesting observation is the apparent species-specific placental ability to preferentially synthesize either cholic acid or chenodeoxycholic acid. It is known that 7α-hydroxycholesterol generated in the process of BA synthesis is epimerized either (i) by HSD3B7/Hsd3b7 to produce 7α-hydroxy-4-cholesten-3-one, which is then converted by CYP8B1/Cyp8b1, followed by the actions of AKR1D1/Akr1d1 and AKR1C4/Akr1c4 on the metabolites to generate cholic acid or (ii) *by* AKR1D1/Akr1d1 and AKR1C4/Akr1c4 to generate chenodeoxycholic acid [1,5]. The expression profiles of *AKR1D1*/*Akr1d1, AKR1C4/Akr1c4*, and *CYP8B1*/*Cyp8b1*, together with those of *CYP27A1/Cyp27a1* and *CYP7A1/Cyp7a1* (Table 1 and Figure 1B and Figure 2A)*,* suggest that the human placenta preferentially synthesizes cholic acid via the alternative pathway. On the other hand, the murine placenta produces predominantly chenodeoxycholic acid through both the classic and alternative pathways, which thereafter is metabolized into muricholic acid. The final steps in BA biogenesis, a process that involves the BAAT/Baat-mediated conjugation of an amino acid (e.g., taurine) [1,2,3], may differ between tissues. *BAAT* is highly expressed in the human liver (Figure 1A), the mouse placenta, and the hypothalamus, but it was not detected in the human placentas (Table 1 and Figure 1B). This finding suggests a species-specific difference in the placental ability to synthesize conjugated BAs. However, it is currently unknown if gestational age plays a major role in the determined gene expression profiles in mouse placentas. Indeed, it cannot be excluded that some of the observed differences in the expression patterns could be related to gestational age differences between murine placentas (collected at E17.5, which is an earlier time point than the human term placentas) and human term placental tissues. Of note, some of the tested enzymes (e.g., 3β-HSDs, 17β-HSDs, and AKR1Cs) are not only part of the BA synthetic machinery but are also involved in the biosynthesis of steroid hormones [23,24]. However, there are also major differences regarding the enzymatic repertoire between these two steroidogenic pathways. CYP11A, for instance, catalyzes the first and rate-limiting step in the synthesis of steroid hormones and their corresponding metabolites (e.g., progesterone, 17-hydroxyprogesterone, estradiol, and androstenedione) but is not involved in the BA synthetic machinery [23,24]. 

**Figure 3:**
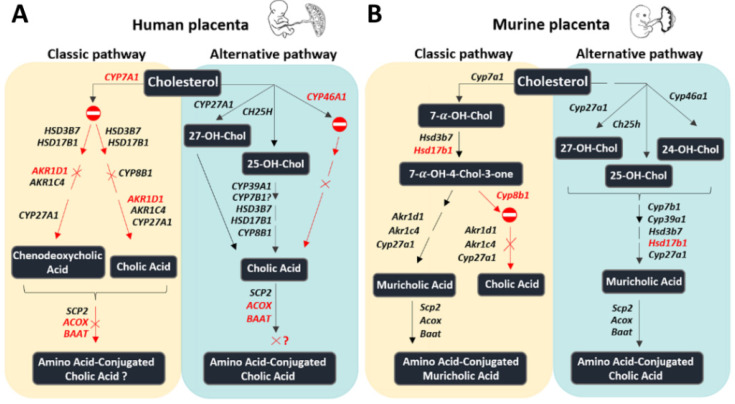
Summary of the proposed bile acid synthetic machinery in the human (**A**) and murine (**B**) placenta. In humans, the only bile acid synthetic mechanisms in the placenta seem to be related to the alternative pathway, initiated by cholesterol 25-hydroxylase and cholesterol 27-hydroxylase, involving, among others, CYP8B1. In contrast, in mice, all enzymes driving the initial steps of the classic and the alternative bile acid biosynthetic pathways are expressed. Red color: the enzyme is absent or detected at negligible levels. The symbol 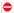 means that the pathway is likely inactive. The symbol *×* means that the downstream product is probably missing. Question mark: pathway where at least one essential enzyme was not detected; Chol: cholesterol.

It is also worth noting that we did not aim at a direct comparison of the expression levels between mouse and human placentas; therefore, they are also illustrated in two different figures (Figure 1B and Figure 2A). However, the overall schematic representation of the BA synthetic machinery in these two species was directly comparable (Figure 3A,B). Moreover, a direct comparison was made regarding the BA synthetic machinery of the two mouse extrahepatic tissues, i.e., the hypothalamus and placenta, since the gene expression in these two tissues was tested at the same developmental stage. The obtained data suggest that BA synthetic pathways are dissimilar between murine extrahepatic tissues (Figure 2A,B).

Concerning the schematic representation of the BA synthetic machinery depicted in Figure 3A, it should be noted that the type of placental cells involved is currently unknown. The data summarized in Table 2 show the gene expression profile of the BA synthetic machinery in isolated human trophoblast cells at two differentiation stages, i.e., cytotrophoblast cells (CTB) and spontaneously differentiated syncytiotrophoblast cells (STB) in comparison to umbilical vein endothelial cells (HUVEC). The data indicate that, unlike our findings in placental tissue, CYP7A1 is detectable in CTB and STB (Table 2). The apparent discrepancy between placental tissues and cell-based data (Figure 3A and Table 2) may result from a dilution effect occurring in placental tissue, which includes a variety of cell types. On the other hand, we did not find CYP7A1 mRNA expression in HUVEC (Table 2). Since CYP7B1 is expressed in placental tissue and not in CTB and STB, we assume that the placental expression results mostly from other cell types. The expression pattern of all the remaining genes detected in the placental tissues was comparable to CTB, STB, and HUVEC (see Table 2 and Figure 3A).

## 3. Materials and Methods

### 3.1. Collection of Human Placenta and Liver Samples

Pregnant women were under obstetrical care at the Division of Gynecology and Obstetrics, Lindenhofgruppe, Bern, Switzerland. Placentas from pregnancies without pathologies and terminated at term (≥37 gestational weeks) were obtained after elective primary cesarean section upon patients’ request or due to breech presentation. Detailed clinical characteristics of the term pregnancies are summarized in Table 3. Human liver tissue served as a positive control tissue for the validation of the primers amplifying BA synthesis-related genes. Liver samples were collected from discarded resected liver tissue from patients undergoing surgical resection in the Department of Visceral Surgery and Medicine. 

### 3.2. Animal Tissue Collection

Animal handling was previously described in detail [20,21]. Briefly, CD1 mice (Harlan Sprague Dawley Inc., Indianapolis, IN, USA) were maintained in a pathogen-free temperature-controlled (22 ± 1 °C) mouse facility on a reverse 12 h light−dark cycle, according to institutional guidelines, with food and water *ad libitum*. Female mice were mated at 11−13 weeks of age, and the presence of a vaginal copulation plug denoted day 0.5 of gestation. On GD17.5, pregnant females were anesthetized with an overdose of ketamine-xylazine (1:1, 20% in saline), and through cesarean section, the placentas were excised, frozen, and processed for RNA extraction. Placental libraries were prepared with the Illumina TruSeq Stranded Total RNA Library Preparation kit with Ribo Zero Gold (Illumina, #RS-122-2301, San Diego, CA, USA) according to the instructions using 1000 ng total RNA as the starting material. RNA-seq was performed on RNA extracted from whole placenta lysates from female ICR/CD1 mice on ED17.5. Data are available in the Sequence Read Archive (SRA) under the BioProject accession number PRJNA434509 and SRA accession number SRP133035. The mouse hypothalamic array was performed on adult nonpregnant females. For these experiments, three-month-old CD1 females were sacrificed, and the hypothalamus was dissected and immediately frozen for later extraction of the total RNA, as previously described [20,21]. Gene expression in the hypothalamus was analyzed using the Agilent microarray kit sureprint G3 and published in previous manuscripts [20,21]. 

### 3.3. Isolation, Characterization, and Culturing of Cells

Isolation of CTB from a healthy human term placenta was performed as previously described [25]. The purity of CTB was determined by cell staining with anti-cytokeratin 7 (CK7; marker of epithelial cells) and anti-vimentin (marker of fibroblast-like cells), followed by a flow cytometry analysis in FACSDiva (BD Biosciences, San Jose, CA, USA), as previously described [25]. Then, CTB of ≥95% purity were seeded at a density of 200,000 cells/cm^2^ in a DMEM high glucose culture medium (Thermo Fisher Scientific, Waltham, MA, USA) supplemented with fetal bovine serum (FBS, 10%) and 100 U/mL penicillin-streptomycin under standard conditions (i.e., 37 °C, 5% CO_2_). Cells were kept in the culture for 24 h (CTB stage) or 72 h to allow spontaneous differentiation (STB stage). The syncytialization process was confirmed by visualization under the microscope and mRNA analysis of syncytialization markers. 

The procedure used for the isolation of HUVEC was previously described [26]. The purity of the isolated HUVEC was assessed by a flow cytometry analysis of cells stained with anti-CD31/PECAM1 (Novus Biologicals, Centennial, CO, USA; positive marker) and anti-fibroblast (ERTR7) (Novus Biologicals, Centennial, CO, USA; negative marker). HUVEC with ≥95% purity were cultured at a density of 20,000 cells/cm^2^ in endothelial cell growth medium (Curio Biotech, Visp, Switzerland) supplemented with 5% FBS and 100 U/mL penicillin–streptomycin under standard conditions. 

CTB, STB, and HUVEC were harvested in a Trizol reagent according to the manufacturer’s instructions (Invitrogen, Paisley, UK) and stored at −80 °C until further analysis.

### 3.4. Quantitative RT-PCR

Total RNA from the primary cells (CTB, STB, and HUVEC) or from approximately 50 mg of human placental tissue was extracted with a Trizol reagent (Invitrogen, UK) following the manufacturer’s instructions. Considering the heterogeneity of placental tissue and our previous findings, which led to a standardization of the placental tissue collection procedure for gene expression analysis [27], we collected each analyzed specimen from the central area of the placenta. 

Concentrations of the extracted total RNA from cells and placental tissues were calculated by measuring the absorbance (A) at 260 nm; purity was assessed by ratios of A260/280 and A260/230 measured on a NanoDrop^TM^ 1000 spectrophotometer (Thermo Fisher Scientific, Waltham, MA, USA). 

Next, 2 μg of total RNA from each specimen were reverse-transcribed in a total volume of 40 μL by using the GoScript™ Reverse Transcriptase System (Promega, Dübendorf, Switzerland) according to the manufacturer’s instructions. RT-PCR was carried out on the CFX384 Touch™ Real-Time PCR Detection System (Bio-Rad Laboratories Inc., Hercules, CA, USA) by using both the SYBR^®^ Green PCR master mix detection kit (Promega, Dübendorf, Switzerland) and a FAM-labeled assay (Life Technologies, Waltham, MA, USA). The placental mRNA abundances of the BA synthesis-related genes listed in Table 4 were analyzed. The primers used for PCR amplification are shown in Table 4.

It is important to highlight that some of the genes constituting the BA synthetic machinery (e.g., those active in the intermediate steps of BA synthesis, such as *AKR1C4, HSD3B7*) are “non-liver-specific”, as they are also active in other tissues and/or play a role in different metabolic processes, such as steroid hormone synthesis. On the other hand, literature data reporting the comparative gene expression profiles of critical enzymes needed for BA synthesis across human tissues or cells are missing. Therefore, it was challenging to identify *a priori* a single negative control (tissue or cell) that would suit all the genes studied in the current study. Nevertheless, we tested the gene expression in five different human cell types: isolated primary HUVEC, Hela cells, HEK295 cells, and human mammary epithelial cells, as well as primary human CTB and STB grown in the same DMEM growth medium. We observed that BA synthesis-related genes showed differential expression across these cell types, underlining the difficulty in selecting one single cell line as the negative control for the entire set of genes tested in this study (data partly shown in Table 2). 

### 3.5. Mathematical Evaluation

The qPCR amplification of the selected BA synthesis-related genes and the reference genes (tyrosine 3-monooxygenase/tryptophan 5-monooxygenase activation protein zeta = *YWHAZ* and *Ubiquitin)* in the human placenta and human liver was assessed by recording the corresponding Ct values. The expression of the measured genes ranged between the Ct values 19.2 (lowest Ct value) and 34.4 (highest Ct value) in the human liver. Thus, the latter value served for the evaluation of gene expression in the placenta and was defined as the cut-off value above which the gene expression (detected in at least 80% of the studied population) was considered as lacking. For each sample, the delta Ct value was calculated as the Ct value of a selected BA-related gene minus the corresponding mean Ct value of *YWHAZ* and *Ubiquitin*. Given the mean Ct values of *YWHAZ* and *Ubiquitin* (used as reference genes) in the human placenta (24.9 ± 2.3) and liver (23.7 ± 0.7), the delta Ct value (0) equals the target Ct value of 24.9 (in the placenta) and 23.7 (in the liver), respectively.

## 4. Conclusions

The data reported in this study demonstrate that the placenta expresses at the mRNA level the necessary machinery allowing for local BA synthesis. Thus, the placenta could not only be an interface for the maternal–fetal exchange of BAs arising from the maternal enterohepatic circulation but also a potential extrahepatic source of BAs. Functionally, BAs are known to modulate diverse important cellular/tissue processes, including proliferation, angiogenesis, apoptosis, energy, and lipid metabolism, which are the processes important for the development of the placenta and a successful pregnancy. Importantly, to further investigate the autocrine, paracrine, and endocrine actions of placentally secreted BAs and their effects on maternal health and disease, the collection of protein and functional data on the de novo synthesis of BAs by the placenta should be acquired from human and murine placental explants and/or isolated placental primary trophoblast cells (e.g., CTB and STB), which are exposed to specific prototypical CYP inducers.

## Figures and Tables

**Table 1:** Expression of bile acid synthesis-related genes in the human placenta and liver.

Genes	Tissue	Detection	Mean ± SD ^a^ Ct Values	Range of Ct Values ^1^
*ACOX2*	Placenta (n = 12)	8/12	35.4 ± 2.82	32.5–39.5
	Liver (n = 3)	3/3	23.8 ± 2.26	21.5–26.0
*AKR1C4*	Placenta (n = 12)	12/12	30.5 ± 2.78	23.2–33.1
	Liver (n = 3)	3/3	19.6 ± 0.55	19.2–20.2
*AKR1D1*	Placenta (n = 12)	5/12	37.8 ± 1.88	34.9–39.9
	Liver (n = 3)	3/3	26.4 ± 6.32	19.5–31.9
*BAAT*	Placenta (n = 12)	1/12	39.5	-
	Liver (n = 3)	3/3	24.7 ± 0.44	24.2–25.0
*CYP7A1*	Placenta (n = 12)	4/12	35.6 ± 2.21	32.3–37.1
	Liver (n = 3)	3/3	26.6 ± 4.63	23.6–31.9
*CYP7B1*	Placenta (n = 12)	11/12	34.6 ± 5.11	20.7–38.7
	Liver (n = 3)	3/3	28.5 ± 1.79	27.5–30.6
*CYP8B1*	Placenta (n = 12)	12/12	33.5 ± 3.46	24.9–38.2
	Liver (n = 3)	3/3	26.7 ± 2.11	24.3–28.0
*CYP27A1*	Placenta (n = 12)	10/12	32.9 ± 3.35	29.7–38.9
	Liver (n = 3)	3/3	21.4 ± 1.30	20.1–22.7
*CYP39A1*	Placenta (n = 12)	12/12	31.1 ± 3.33	22.6–35.3
	Liver (n = 3)	3/3	22.7 ± 1.03	21.6–23.6
*CYP46A1*	Placenta (n = 12)	5/12	38.4 ± 0.75	37.7–39.5
	Liver (n = 3)	3/3	31.5 ± 2.18	29.6–33.9
*CH25H*	Placenta (n = 12)	12/12	31.7 ± 3.44	23.7–37.7
	Liver (n = 3)	3/3	29.4 ± 1.78	28.1–30.4
*HSD3B7*	Placenta (n = 12)	10/12	33.7 ± 3.94	28.9–39.7
	Liver (n = 3)	3/3	25.4 ± 2.35	23.1–27.8
*HSD17B1*	Placenta (n = 12)	12/12	25.5 ± 4.78	21.3–37.0
	Liver (n = 3)	3/3	32.7 ± 2.94	29.3–34.4
*SLC27A2*	Placenta (n = 12)	11/12	32.3 ± 4.29	26.9–38.9
	Liver (n = 3)	3/3	22.0 ± 1.26	20.7–23.2
*SCP2*	Placenta (n = 12)	12/12	29.9 ± 4.07	19.7–34.7
	Liver (n = 3)	3/3	25.8 ± 3.13	22.2–27.9
*YWHAZ*	Placenta (n = 12)	12/12	26.5 ± 3.11	20.9–30.4
	Liver (n = 3)	3/3	24.1 ± 0.7	23.0–24.7
*Ubiquitin*	Placenta (n = 12)	12/12	23.4 ± 2.15	20.0–27.0
	Liver (n = 3)	3/3	23.3 ± 0.77	22.4–23.9

^1^ Ct value ranges show the minimum and maximum Ct values. The respective gene was considered as being expressed in the placenta when its expression was detected in at least 80% of all tested placental samples with a mean Ct value ≤ 34.4. This threshold value corresponds to the highest Ct value for a bile acid synthesis-related gene detected in the positive tissue (i.e., liver). Therefore, to assess the gene expression in the human placenta, this Ct value served as a cut-off value above which the gene expression was considered lacking. Since the gene expression analysis in liver tissues was performed using 25 ng of cDNA, the same concentration was applied to the placental samples for comparative purposes. ^a^ SD: the standard deviation is shown when applicable.

**Table 2:** Gene expression of bile acid synthesis-related genes in human primary trophoblasts and human umbilical vein endothelial cells.

Genes	Primary Cytotrophoblasts (CTB; n = 5)	Primary Syncytiotrophoblasts (STB; n = 5)	Human Umbilical Vein Endothelial Cells (HUVEC; n = 2)
*CYP7A1* CT value Similarity to placental tissue	31.0 ± 1.26 No	31.4 ± 1.30 No	n/d Yes
*CYP27A1* CT value Similarity to placental tissue	29.1 ± 2.47 Yes	28.2 ± 4.68 Yes	28.3 ± 0.06 Yes
*CYP46A1* CT value Similarity to placental tissue	n/d Yes	n/d Yes	n/d Yes
*CH25H* CT value Similarity to placental tissue	29.3 ± 2.69 Yes	28.7 ± 2.63 Yes	32.9 ± 1.02 Yes
*CYP39A1* CT value Similarity to placental tissue	25.9 ± 1.03 Yes	24.8 ± 1.84 Yes	30.3 ± 0.83 Yes
*CYP7B1* CT value Similarity to placental tissue	n/d No	n/d No	n/d No
*CYP8B1* CT value Similarity to placental tissue	31.2 ± 1.70 Yes	30.2 ± 2.03 Yes	35.2 ± 0.72 Yes
*HSD3B7* CT value Similarity to placental tissue	28.4 ± 0.18 Yes	29.0 ± 0.33 Yes	26.3 ± 0.44 Yes
*HSD17B1* CT value Similarity to placental tissue	24.3 ± 3.73 Yes	25.6 ± 4.35 Yes	28.1 ± 3.92 Yes
*ACOX2* CT value Similarity to placental tissue	n/d Yes	n/d Yes	n/d Yes
*AKR1D1* CT value Similarity to placental tissue	26.9 ± 1.65 No	25.7 ± 1.85 No	32.4 ± 1.90 No
*AKR1C4* CT value Similarity to placental tissue	24.0 ± 1.63 Yes	24.1 ± 1.53 Yes	32.9 ± 3.65 Yes
*SLC27A2* CT value Similarity to placental tissue	24.5 ± 1.11 Yes	24.6 ± 0.59 Yes	34.9 ± 0.21 Yes
*BAAT* CT value Similarity to placental tissue	n/d Yes	n/d Yes	n/d Yes
*SCP2* CT value Similarity to placental tissue	21.8 ± 1.51 Yes	21.5 ± 1.50 Yes	33.8 ± 0.19 Yes
*YWHAZ* CT value Similarity to placental tissue	21.8 ± 2.66 Yes	21.9 ± 1.19 Yes	22.5 ± 0.21 Yes
*Ubiquitin* CT value Similarity to placental tissue	24.3 ± 2.63 Yes	25.3 ± 1.89 Yes	19.5 ± 0.1 Yes

Data show the mean Ct ± SD of RT-qPCR measurements from five primary cell isolations of trophoblasts and two primary cell isolations of HUVEC, respectively. Yes: indicates a similar expression pattern compared to placental tissue, regardless of the actual Ct values (i.e., in both cases, the genes are either detected or not detected). No: indicates a different expression profile compared to the placental tissue. Since the gene expression analysis on the placental tissues was performed using 25 ng of cDNA, the same concentration was applied in the placental cells. CTB: primary cytotrophoblast cells; STB: primary syncytiotrophoblast cells, resulting from in vitro spontaneously differentiated CTB; HUVEC: human umbilical vein endothelial cells; n/d; not detected.

**Table 3:** Clinical characteristics of the studied human term pregnancies (n = 12).

Parameter	Clinical Data		
Gravidity	Median = 2	25–75% = 1.5–2.5	Range 1–3
Parity	Median = 2	25–75% = 1–2.5	Range 1–3
Gestational age (weeks)	Mean = 39.4	SD = 0.9	Range 38–41
Maternal age (years)	Mean = 32.6	SD = 4.6	Range 27–38
Smoking/drugs	0/12		
Birth weight (gram)	Mean = 3525	SD = 211	Range 3300–3736
Placental weight (gram)	Mean = 649	SD = 155	Range 500–800
Sex offspring	Male = 6	Female = 6	

**Table 4:** Primers used for the amplification of BA synthesis-related genes in human tissues.

Gene	Forward Primer (5′-> 3′)	Reverse Primer (5′-> 3′)	Acc. Number
*ACOX2*	CCGCAGGAAAGTTGAGAGCA	AAGGCCACGTCTCCAGAAAG	NM_003500.4
*AKR1C4*	AGATGGTCCAACCAGCCTTG	TGGCTTGAGAGCCATTGGG	NM_001818.5
*AKR1D1*	CAAACACAAGCCAGTCAGCAAC	GATGATCGCGCCACATGAGC	NM_005989.4
*BAAT*	GTTCTCTTGCTAGGTTTTG	ACCATCTGAAAGGGAATC	NM_001701.4
*CYP7A1*	TTGCTACTTCTGCGAAGGCA	TCCGTGAGGGAATTCAAGGC	NM_000780.4
*CYP7B1*	GTTCTTCTTGGTGGAAAG	ATTGATAGCAGAGGTGAA	NM_004820.5
*CYP8B1*	TACACTCAGCCAGCACCAAG	AAAGAGGCTGTCCTCATGCC	NM_004391.3
*CYP27A1*	CGGTCCAATGTGGATGTCCT	GTACCAGTGGTGTCCTTCCG	NM_000784.4
*CYP39A1*	TCGTACAGCATCAATTCCAAAGA	CCATTGTCCCATGAGTGCCT	NM_016593.5
*CYP46A1*	AGGTCATTGGTTCTAAGAG	ATAGGTGCTGAACAAGAG	NM_006668.2
*CH25H*	ACATACGTGGGCTTTTGCCT	CATGCTGGTAGAGGGTCTGC	NM_003956.4
*HSD3B7*	GTCTATGTGGGCAATGTT	TCCATCGTAGCAGAAGTA	NM_025193.4
*HSD17B1*	TTATTGCGCCAGCAAGTTCG	TTCTCCATGAAGGCGGTGTG	NM_000413.4
*SLC27A2*	ACCACAGGTCTTCCAAAAGCA	AGTCCGCAAGGCAAGAGTAG	NM_003645.4
*SCP_2_*	TTAGATAAGAAGGCTGACTG	TACTTACCGACTGAGGAT	AH004933.2
*YWHAZ* ^a^	CCGTTACTTGGCTGAGGTTG	AGTTAAGGGCCAGACCCAGT	NM_001135699.2
*Ubiquitin*	TCGCAGCCGGGATTTG	GCATTGTCAAGTGACGATCACA	NM_021009

The primers used in this study were designed by using Beacon Designer™ software, version 8.21, except the primers marked with a superscript letter ^a^, which were taken from a previous study. Quantitative RT-PCR was carried out using the CFX384 Touch™ Real-Time PCR Detection System (Bio-Rad Laboratories Inc., USA). All primers were validated in human livers.

## Data Availability

The original contributions presented in the study are included in the article, further inquiries can be directed to the corresponding author.

## References

[1] Russell D.W. (2003). The enzymes, regulation, and genetics of bile acid synthesis. Annu. Rev. Biochem..

[2] Russell D.W., Setchell K.D.R. (1992). Bile acid biosynthesis. Biochemistry.

[3] Chiang J.Y.L. (2009). Bile acids: Regulation of synthesis. J. Lipid Res..

[4] Hofmann A.F. (2009). The enterohepatic circulation of bile acids in mammals: Form and functions. Front. Biosci.-Landmark.

[5] Monteiro-Cardoso V.F., Corlianò M., Singaraja R.R. (2021). Bile Acids: A Communication Channel in the Gut-Brain Axis. NeuroMolecular Med..

[6] Pan X., Elliott C.T., McGuinness B., Passmore P., Kehoe P.G., Hölscher C., McClean P.L., Graham S.F., Green B.D. (2017). Metabolomic profiling of bile acids in clinical and experimental samples of Alzheimer’s disease. Metabolites.

[7] Milona A., Owen B.M., Cobbold J.F.L., Willemsen E.C.L., Cox I.J., Boudjelal M., Cairns W., Schoonjans K., Taylor-Robinson S.D., Klomp L.W.J. (2010). Raised hepatic bile acid concentrations during pregnancy in mice are associated with reduced farnesoid X receptor function. Hepatology.

[8] Castaño G., Lucangioli S., Sookoian S., Mesquida M., Lemberg A., Di Scala M., Franchi P., Carducci C., Tripodi V. (2006). Bile acid profiles by capillary electrophoresis in intrahepatic cholestasis of pregnancy. Clin. Sci..

[9] Egan N., Bartels Ä., Khashan A.S., Broadhurst D.I., Joyce C., O’Mullane J., O’Donoghue K. (2012). Reference standard for serum bile acids in pregnancy. BJOG Int. J. Obstet. Gynaecol..

[10] Colombo C., Roda A., Roda E., Buscaglia M., Dell’Agnola C.A., Filippetti P., Ronchi M., Sereni F. (1985). Correlation between fetal and maternal serum bile acid concentration. Pediatr. Res..

[11] Sasaki H. (1984). Development of Bile Acid Metabolism in Neonates during Perinatal Period Part 1: Bile acid levels in sera of Mothers, fetuses and neonates. Pediatr. Int..

[12] Itoh S., Onishi S., Isobe K., Manabe M., Inukai K. (1982). Foetomaternal relationships of serum bile acid pattern estimated by high-pressure liquid chromatography. Biochem. J..

[13] Geenes V., Lövgren-Sandblom A., Benthin L., Lawrence D., Chambers J., Gurung V., Thornton J., Chappell L., Khan E., Dixon P. (2014). The Reversed Feto-Maternal Bile Acid Gradient in Intrahepatic Cholestasis of Pregnancy Is Corrected by Ursodeoxycholic Acid. PLoS ONE.

[14] Ontsouka E., Epstein A., Kallol S., Zaugg J., Baumann M., Schneider H., Albrecht C. (2021). Placental expression of bile acid transporters in intrahepatic cholestasis of pregnancy. Int. J. Mol. Sci..

[15] Sepúlveda W.H., González C., Cruz M.A., Rudolph M.I. (1991). Vasoconstrictive effect of bile acids on isolated human placental chorionic veins. Eur. J. Obstet. Gynecol. Reprod. Biol..

[16] Lofthouse E.M., Torrens C., Manousopoulou A., Nahar M., Cleal J.K., O’Kelly M.I., Sengers B.G., Garbis S.D., Lewis R.M. (2019). Ursodeoxycholic acid inhibits uptake and vasoconstrictor effects of taurocholate in human placenta. FASEB J..

[17] De Aguiar Vallim T.Q., Tarling E.J., Edwards P.A. (2013). Pleiotropic roles of bile acids in metabolism. Cell Metab..

[18] Li S., Jia Z.H., Wei X.R., Ma S., Lu T.C., Li T.T., Gu Y.Y. (2020). Role of bile acid on maintaining metabolic homeostasis. J. Shanghai Jiaotong Univ. Med. Sci..

[19] Kawamata Y., Fujii R., Hosoya M., Harada M., Yoshida H., Miwa M., Fukusumi S., Habata Y., Itoh T., Shintani Y. (2003). A G protein-coupled receptor responsive to bile acids. J. Biol. Chem..

[20] Schroeder M., Jakovcevski M., Polacheck T., Drori Y., Luoni A., Röh S., Zaugg J., Ben-Dor S., Albrecht C., Chen A. (2018). Placental miR-340 mediates vulnerability to activity based anorexia in mice. Nat. Commun..

[21] Schroeder M., Jakovcevski M., Polacheck T., Drori Y., Ben-Dor S., Röh S., Chen A. (2018). Sex dependent impact of gestational stress on predisposition to eating disorders and metabolic disease. Mol. Metab..

[22] Fisher M.M., Yousef I.M. (1973). Sex differences in the bile acid composition of human bile: Studies in patients with and without gallstones. Can. Med. Assoc. J..

[23] Payne A.H., Hales D.B. (2004). Overview of steroidogenic enzymes in the pathway from cholesterol to active steroid hormones. Endocr. Rev..

[24] Karahoda R., Kallol S., Groessl M., Ontsouka E., Anderle P., Fluck C., Staud F., Albrecht C. (2021). Revisiting steroidogenic pathways in the human placenta and primary human trophoblast cells. Int. J. Mol. Sci..

[25] Fuenzalida B., Cantin C., Kallol S., Carvajal L., Pastén V., Contreras-Duarte S., Albrecht C., Gutierrez J., Leiva A. (2020). Cholesterol uptake and efflux are impaired in human trophoblast cells from pregnancies with maternal supraphysiological hypercholesterolemia. Sci. Rep..

[26] Guzmán-Gutiérrez E., Westermeier F., Salomón C., González M., Pardo F., Leiva A., Sobrevia L. (2012). Insulin-increased L-arginine transport requires A2A adenosine receptors activation in human umbilical vein endothelium. PLoS ONE.

[27] Huang X., Baumann M., Nikitina L., Wenger F., Surbek D., Körner M., Albrecht C. (2013). RNA degradation differentially affects quantitative mRNA measurements of endogenous reference genes in human placenta. Placenta.

